# Supplementary material for: Astrocytes modulate neuronal development by S100A6 signaling
Source: Nat Commun. 2025 Oct 13;16:9049. doi: 10.1038/s41467-025-64405-y (PMC12518605; doi:10.1038/s41467-025-64405-y)

Supplementary Information to the manuscript:

Astrocytes modulate neuronal development by S100A6 signaling

Valentina Cinquina[1], Evgenii O. Tretiakov[1], Predrag Kalaba[2], Alán Alpár[3,4], Daniela Calvigioni[1,11], Fabiana Piscitelli[5], Erik Keimpema[1], Vincenzo Di Marzo[5,6,7], Alexej Verkhratsky[8,9] and Tibor Harkany[1,10,§]

[1]Department of Molecular Neurosciences, Center for Brain Research, Medical University of Vienna, Vienna, Austria; [2]Institute of Biological Chemistry, Faculty of Chemistry, University of Vienna, Vienna, Austria; [3]SE NAP Research Group of Experimental Neuroanatomy, Semmelweis University, Budapest, Hungary; [4]Developmental Biology, Department of Anatomy, Histology, and Embryology, Semmelweis University, Budapest, Hungary; [5]Endocannabinoid Research Group, Institute of Biomolecular Chemistry (ICB), National Research Council (CNR), Pozzuoli, Italy; [6]Canada Excellence Research Chair on the Microbiome-Endocannabinoidome Axis in Metabolic Health, Institut Universitaire de Cardiologie et de Pneumologie de Québec, Université Laval, Québec, QC, Canada; [7]Institut sur la Nutrition et les Aliments Fonctionnels, Centre Nutriss, Université Laval, Québec, QC, Canada; [8]Faculty of Biology, Medicine and Health, The University of Manchester, Manchester, United Kingdom; [9]Achucarro Centre for Neuroscience, IKERBASQUE, Basque Foundation for Science, Bilbao, Spain; [10]Department of Neuroscience, Biomedicum 7D, Karolinska Institutet, Solna, Sweden.

[11]*Present address*: Department of Neuroscience, Biomedicum 4B, Karolinska Institutet, Solna, Sweden.

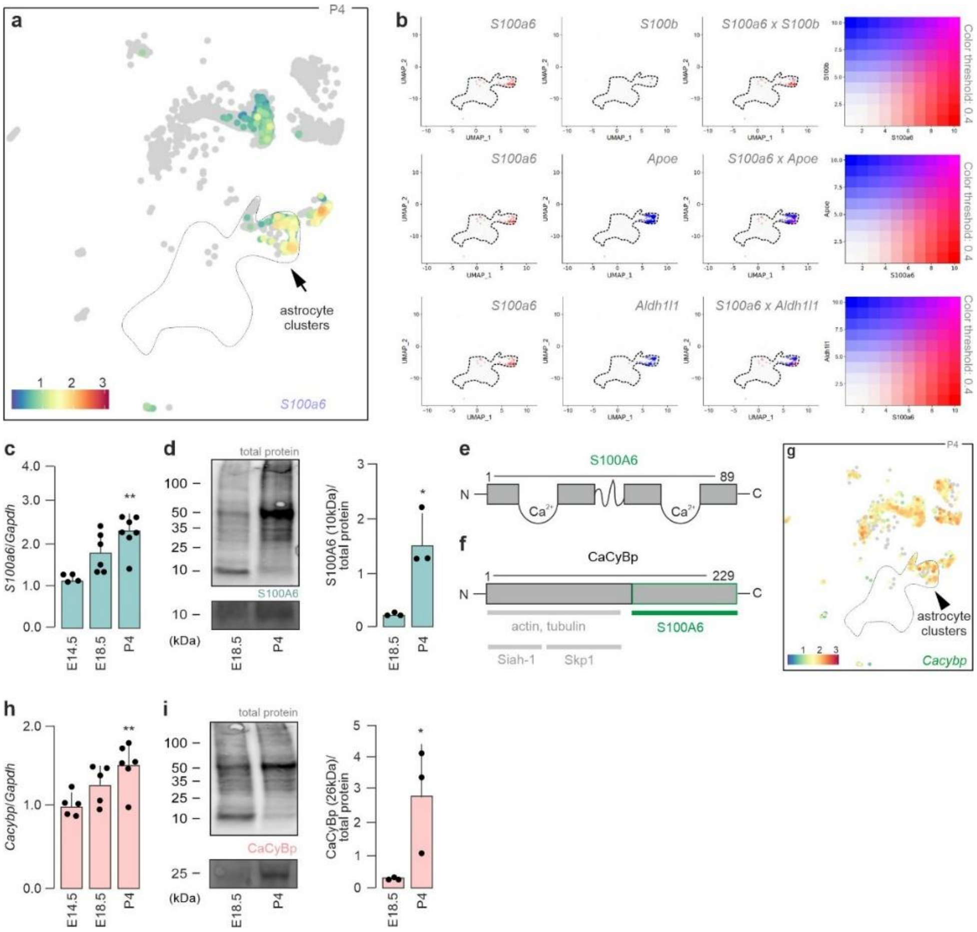

Supplementary Figure 1. S100A6 and CaCyBp in the developing mouse brain. a. UMAP plot showing the distribution of *S100a6* expression on postnatal day 4 (P4). **b.** UMAP plot showing the distribution of *S100a6*$^+$/*S100b*$^+$, *S100a6*$^+$/*Apoa*$^+$, and *S100a6*$^+$/*Aldh1l1*$^+$ astrocytes. **c**. *S100a6* expression (mRNA) during fetal brain development, quantified by qPCR and normalized to *Gapdh* as a housekeeping standard from *n* = 4 male mice/E14.5; *n* = 6 male mice/E18.5; *n* = 7 male mice/P4; (***p* = 0.003 for E14.5 *vs*. P4, one-way ANOVA followed by Tukey's multiple comparisons; means ± s.d). **d**. Western analysis of S100A6 protein levels during pre- and neonatal life. Data were normalized to the total protein content of the samples (means ± s.d from *n* = 3 male mice/time point; **p* = 0.017 for E18.5 *vs*. P4, two-tailed Student's *t*-test). **e**. Schematic representation of S100A6 domains (residues 1-89). An S100A6 monomer consists of two EF-hand domains, which are the structural motifs for Ca^{2+} binding. Figure was adapted from Ref.[1] under a CC BY 4.0 licence (https://creativecommons.org/licenses/by/4.0/). **f**. Schematic representation of CaCaCyp (residues 1-229), including effector protein binding sites, reprinted from Ref.[2]with permission from Elsevier. **g.** UMAP plot of *Cacybp* expression on P4. **h**. *Cacybp* mRNA expression during fetal brain development, normalized to *Gapdh* (means ± s.d*; n* = 5 male mice/E14.5 and E18.5; *n* = 6 male mice/P4; ***p* = 0.009 for E14.5 *vs*. P4, one-way ANOVA followed by Tukey's multiple comparisons). **i**. CaCyBp protein levels in the mouse cerebral cortex at the ages indicated (*n* = 3 male mice/time point, **p* = 0.0497 for E18.5 *vs*. P4, two-tailed Student's *t*-test). Data were expressed as means ± s.d. throughout. Primary experimental data were included in the 'Source Data File'.

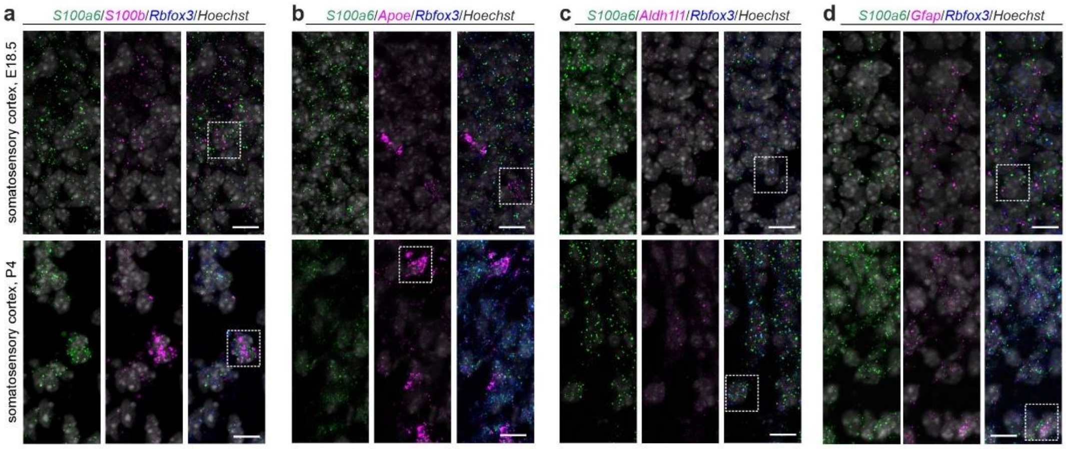

Supplementary Figure 2. **Localization of *S100a6* mRNA in cortical astrocytes.** Overlapping expression of *S100a6* with *S100b* (**a**), *Apoe* (**b**), *Aldh1l1* (**c**), and *Gfap* (**d**) mRNA transcripts in the somatosensory cortex at fetal (E18.5) and neonatal (P4) ages. Note the mutually exclusive pattern between mRNA expression for *S100a6* and *Rbfox3*, the latter encoding neuron-specific nuclear protein (NeuN). Figures are representative from n = 3 male mice/time point. Dashed rectangles denote the location of high-resolution images in Figure 2. *Scale bars* = 10 μm.

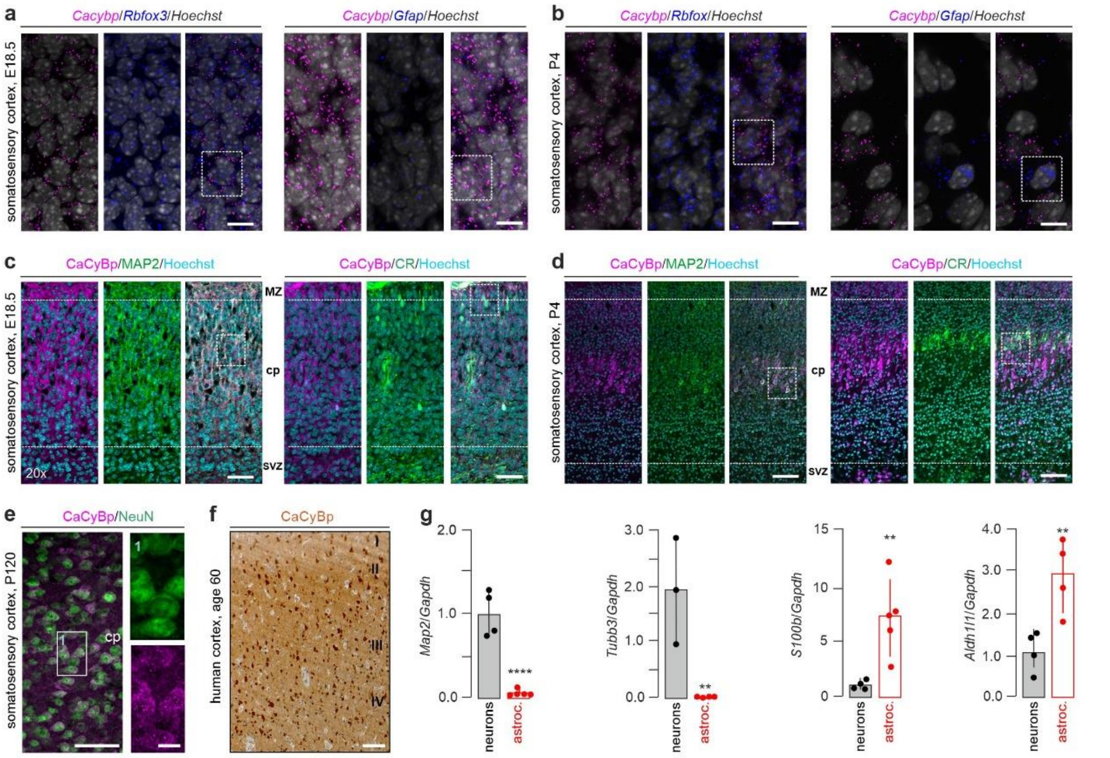

Supplementary Figure 3. **CaCyBp localization in cortical neurons. a,b**. Overlap between the expression of *Cacybp and Rbfox3* (encoding NeuN) transcripts in fetal (E18.5; **a**) and neonatal (P4; **b**) mouse cortices (representative images from *n* = 3 male mice/time point; *scale bars* = 10 μm). **c,d.** Representative images of CaCyBp localization by immunohistochemistry in the somatosensory cortex of fetal (E18.5; **c**) and neonatal (P4, **d**) mice co-labelled for either microtubule-associated protein 2 (MAP2) or calretinin (CR; representative images from *n* = 3 male mice/time point; *scale bars* = 50 μm). Dashed boxes in panels (**a-d**) denote the localization of insets in Figure 2c,f. **e**. Neuronal localization of CaCyBp in the adult mouse cerebral cortex (P120) through co-labelling for NeuN (representative data from *n* = 3 male mice/time point; *scale bar* = 60 μm). Open rectangle ('1') denotes the localization of insets (*scale bar* = 10 μm). **f.** CaCyBp localization to the somatodendritic axis of cortical neuron-like cells in the adult human brain (60 years of age; male subject; *scale bar* = 250 μm). **g**. mRNA levels for *Map2* (****$p < 0.0001$), *Tubb3* (**$p = 0.003$), *S100b* (**$p = 0.007$), and *Aldh1l1* (**$p = 0.009$; all with two-tailed Student's *t*-test) in cultured neurons *vs*. astrocytes harvested from *n* = 4 biological replicates [neurons]; *n* = 5 biological replicates [astrocytes]; both mixed for sex. Data were expressed as means ± s.d. mRNA expression was normalized to *Gapdh*, a housekeeping gene. *Abbreviations*: I-IV, cortical layers 1-4; astroc., astrocytes; cp, cortical plate; MZ, marginal zone; SVZ, subventricular zone. Unprocessed data and statistics were included in the 'Source Data File'.

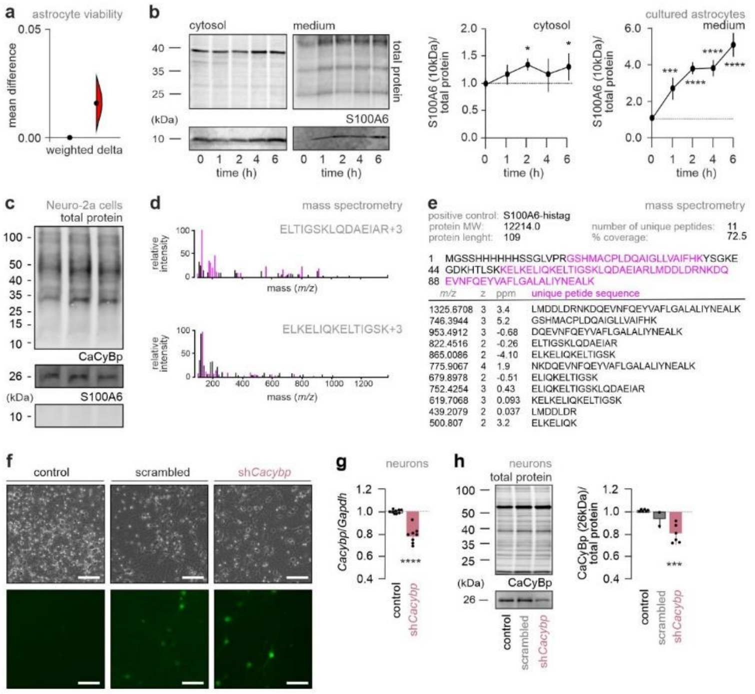

Supplementary Figure 4. **Manipulation of S100A6 content and validation of knock-down experiments**. **a**. MTT assay showing the lack of cell death amongst cultured astrocytes upon glutamate treatment (100 μM; at 24 h). Floating plots show the overall weighted mean difference for all experiments (means ± s.d from *n* = 8 biological replicates; mixed for sex; *see Methods*). Solid circles represent the point estimate of the mean difference, with vertical lines indicating 95% confidence intervals. The shaded curve represents the resampled distribution of the effect size. **b**. S100A6 in the cytosol of astrocytes (*left*) and cell-free culture medium (*right)* after treatment with glutamate (100 μM) for 24 h. Quantitative data were from quadruple experiments; cytosol: **p* = 0.021 [2 h] *vs*. [0 h]; **p* = 0.021 [6 h] *vs*. [0 h]; medium: ****p* < 0.001 [1 h] *vs*. [0 h]; *****p* < 0.0001 [2,4,6 h] *vs*. [0 h]; one-way ANOVA followed by Bonferroni's multiple comparisons. **c**. Western analysis of CaCyBp and S100A6 protein expression in Neuro-2a cells. **d**. Two unique S100A6 peptide fragments were identified by means of LC-MS/MS after exposing Neuro-2a cells to recombinant S100A6 protein (1 μg/ml) for 24 h. **e**. Detectability of recombinant S100A6 protein was analyzed under identical conditions to allow for comparisons. The mass-to-charge ratio (m/z), charge state (z), error in parts per million (ppm), unique peptide sequences, and sequence coverage were provided. **f**. Representative images of cortical neurons transfected with either scrambled shRNA (control) or shRNA against CaCyBp (sh*Cacybp*) at 15 DIV. Constructs were tagged with green fluorescent protein to validate transfection efficacy (*bottom row*). *Scale bars* = 200 μm. **g**. mRNA levels of *Cacybp* in cortical neurons transfected with sh*Cacybp* and cultured for 15 DIV. mRNA content was quantified by qPCR and normalized to *Gapdh* (*****p* < 0.0001, two-tailed Student's *t*-test; *n* = 9 independent experiments.) **h.** Western analysis of CaCyBp protein content from the same experiments. ****p* = 0.0002 (one-way ANOVA following by Bonferroni's multiple caparisons; *n* = 6 independent experiments). Data were expressed as means ± s.d. throughout. Unprocessed data and statistics were included in the 'Source Data File'.

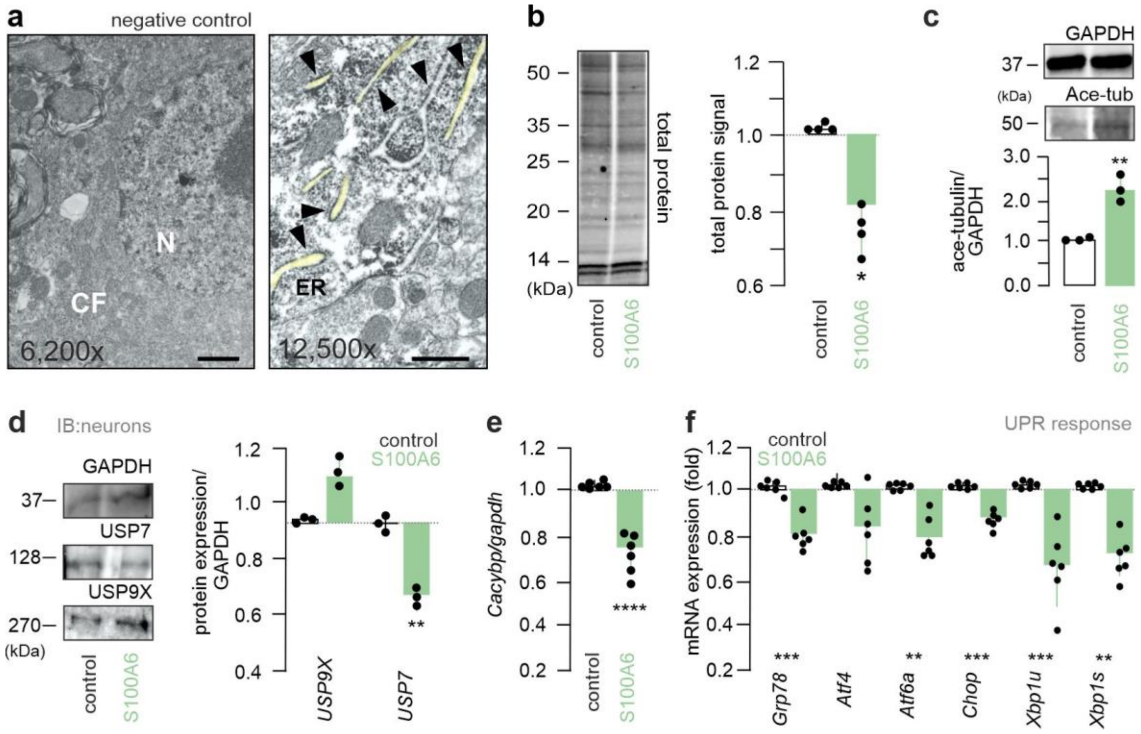

Supplementary Figure 5. **Recombinant S100A6 affects the UPR machinery. a**. Negative control, and CaCyBp localization in the vicinity of the endoplasmic reticulum (ER) of neurons in the dentate gyrus of the adult mouse brain (*black arrowheads*) as revealed by electron microscopy. *Scale bars* = 500 nm (12,500x) and 1 µm (6,200x). *Abbreviations*: CF, cytosolic fraction; N, nuclei. **b.** Total protein content after exposure to recombinant S100A6 (1 µg/µl) for 24 h *in vitro* (*n* = 4 biological replicates; mixed for sex; **p* = 0.04 *vs.* [control]) statistically evaluated by two-tailed Student's *t*-test. **c**. Western blot detection of acetylated tubulin (50 kDa) in cortical neurons after treatment with recombinant S100A6 for 24 h (*n = 3* biological replicates; mixed for sex; ***p* = 0.001 *vs.* [control]). Data were normalized to GAPDH and statistically evaluated by two-tailed Student's *t*-test. **d**. Western blot detection of ubiquitin-specific protease 7 (USP7; 128 kDa; *n = 3* biological replicates; mixed for sex; ***p* = 0.001 *vs.* [control]) and ubiquitin-specific protease 9X (USP9X; 270 kDa; *n = 3* biological replicates; mixed for sex) in cortical neurons exposed to recombinant S100A6. Data were normalized to GAPDH and statistically evaluated by two-tailed Student's *t*-test. **e**. mRNA levels for *Cacybp* (*****p* < 0.0001 *vs.* [control]; *n = 6* biological replicates; mixed for sex) in cortical neurons (E18.5) after S100A6 exposure (1 µg/µl) for 24 h; statistically evaluated by two-tailed Student's *t*-test. **f.** *Grp78* (****p* < 0.001), *Atf4*, *Atf6a* (***p* = 0.002), *Chop* (****p* < 0.001), *Xbp1u* (****p* < 0.001), and *Xbp1s* (***p* = 0.0013) mRNA levels in cortical neurons after exposure to recombinant S100A6 for 24 h. Data were normalized to *Gapdh* (*n* = 6 independent experiments; two-tailed Student's *t*-test) Data were expressed as means ± s.d. throughout. Unprocessed data and statistics were included in the Source Data File.

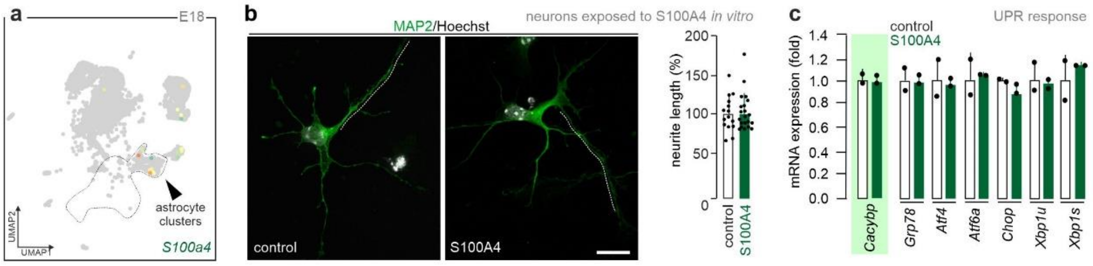

Supplementary Figure 6. **Recombinant S100A4 lacks an effect on either neuronal growth or the UPR machinery. a.** UMAP plot of *S100a4* mRNA expression on E18. **b**. Neuritogenesis of cortical neurons (harvested at P0) was not reduced after exposure to recombinant S100A4 protein (1 μg/μl) for 24 h (*scale bar* = 12 μm). Primary neurite length (μm) was normalized to mock-transfected controls, and expressed as percentage (*n* = 16-21 cells/treatment; mixed for sex). **c**. mRNA levels for *Cacybp, Grp78*, *Atf4*, *Chop, Xbp1u*, and *Xbp1s* in cortical neurons after exposure to recombinant S100A4 (1 μg/μl) for 24 h. Data were normalized to *Gapdh*. Data were expressed as means ± s.d. from duplicate experiments and statistically evaluated by two-tailed Student's *t*-test. Unprocessed data and statistics were included in the Source Data File.

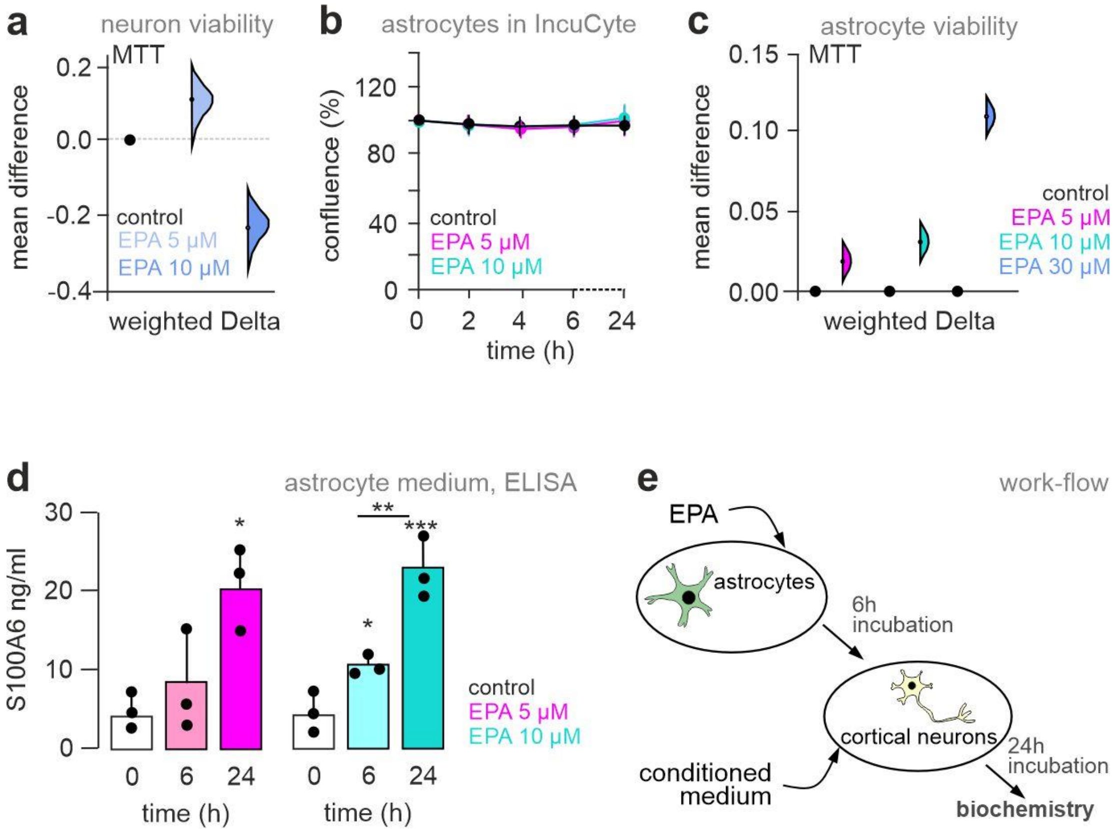

Supplementary Figure 7. **Eicosapentaenoic acid affects astrocyte viability and S100A6 release. a**. MTT assay showing neuronal viability upon EPA treatment (1 μM and 5 μM; at 7 DIV). Floating plots show the overall weighted mean difference for all experiments (*n* = 8 biological replicates; mixed for sex; *see Methods*). Solid circles represent the point estimate of the mean difference, with vertical lines indicating 95% confidence intervals. The shaded curve corresponds to the resampled distribution of the effect size. **b**. Cultured astrocytes were stimulated with either 5 μM (**a**) or 10 μM EPA (**a$_1$**) and compared to vehicle-treated controls. Data were normalized to cell density prior to EPA exposure on day 1 (*n* = 6 independent experiments; mixed for sex). **c**. Astrocyte viability (MTT assay) after EPA stimulation (5 μM, 10 μM, or 30 μM) for 24 h. Floating plots demonstrate the overall weighted mean difference for all experiments (*n* = 6 independent experiment; mixed for sex). Circles represent the point estimate of the mean difference, with vertical lines indicating 95% confidence intervals. The shaded curve represents the resampled distribution of the effect size. **d**. ELISA of S100A6 in culture media upon either 5 μM or 10 μM EPA stimulation for 24 h (*n* = 3 biological replicates; mixed for sex; **p* = 0.015 [0 h] *vs*. [EPA (5 μM) at 24 h], **p* = 0.035 [0 h] *vs*. [EPA (10 μM) at 6 h]; ****p* = 0.035 [0 h] *vs*. [EPA (10 μM) at 24 h]; ***p* = 0.002 [6 h] *vs*. [24 h] (EPA 10 μM); one-way ANOVA followed by Tukey's multiple comparisons). **e**. Schema of co-culture experiments. Data were expressed as means ± s.d. where relevant. Raw data and statistics were included in the Source Data File.

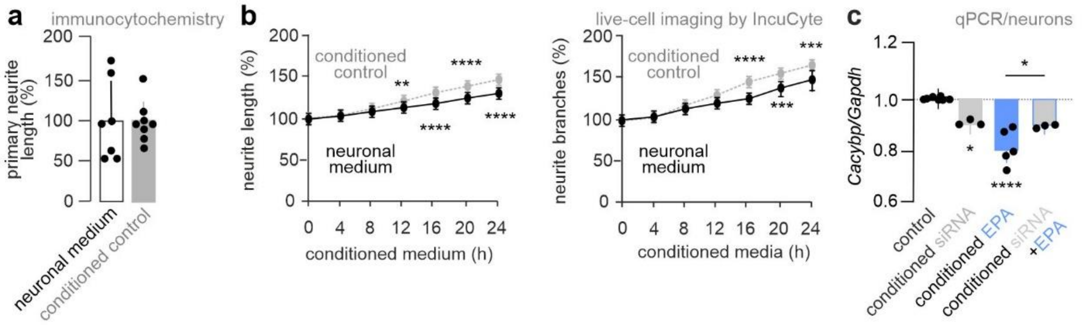

Supplementary Figure 8. **Conditioned astrocyte medium affects neurite outgrowth. a**. Primary neurite length was unchanged 24 h after exposure either to neuronal media (Neurobasal/B27) or astrocyte-conditioned medium. Primary neurite length was normalized to neurons in Neurobasal/B27, and expressed as percentages (*n* = 7 cells in neurobasal; *n* = 7 cells in conditioned medium; mixed for sex). **b.** Neuritogenesis in cortical neurons (E18.5) grown in Neurobasal/B27 or medium pre-conditioned by astrocytes, and monitored by dynamic live-cell imaging (IncuCyte) for 24 h. Data were normalized to neurite length prior to exposure to astrocyte-conditioned medium. Neurite outgrowth was determined by using a manual overlay of each neuron (data from *n* = 8 independent experiments; statistical evaluation: *neurite length*: $**p = 0.014$; $****p < 0.0001$; *neurite branches*: $***p = 0.0001$; $****p < 0.0001$ [all parameters *vs.* neuronal media]). **c**. mRNA levels for *Cacybp* in cortical neurons after exposure to conditioned medium from astrocytes for 24 h. Data were normalized to *Gapdh* from *n* = 3-5 independent experiments ($*p = 0.02$ [cond. siRNA] *vs.* [control]; $****p < 0.0001$ [cond. EPA] *vs.* [control]; $*p = 0.0224$ [cond. EPA] *vs* [cond. siRNA + EPA] multiple comparisons after ANOVA). Data were expressed as means ± s.d. throughout. Unprocessed data and detailed statistics were included in the Source Data File.

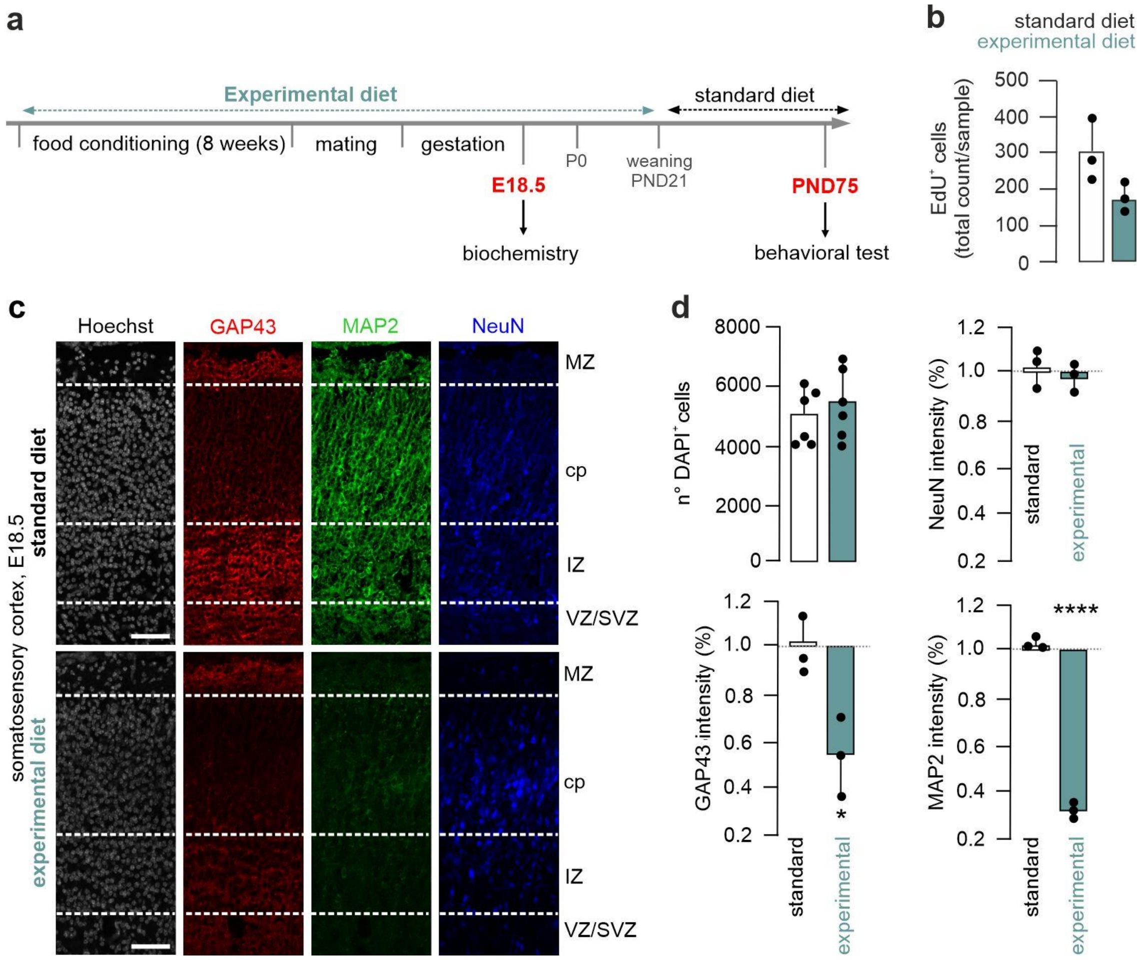

Supplementary Figure 9. Disrupted corticogenesis in embryos exposed to an experimental diet rich in ω-3 polyunsaturated fatty acids. a. Schema of diet administration to C57Bl6/J dams. Administration of the experimental diet commenced eight weeks before gestation, and continued throughout gestation, and lactation. **b**. Quantification of EdU^+ cell numbers in cortical layers of male embryos at E18.5. Cell counting was performed on equal cortical tissue surfaces, and expressed as absolute numbers (n = 3 male mice/group). **c**. Immunohistochemical detection of GAP43, MAP2, and NeuN in the somatosensory cortex of fetal mice (E18.5) after exposure to a standard (*upper*) *vs*. experimental diet (*bottom*; n = 3 male mice/group from independent pregnancies). **d**. Immunoreactivity for GAP43 and MAP2 in E18.5 mouse cortices after experimental diet exposure was reduced by ~65% and ~45%, respectively, as compared to specimens treated with a standard diet. Neither $DAPI^+$ cell numbers nor NeuN immunoreactivity changed (n = 3 male mice/group; *$p = 0.018$ *vs.* [standard diet]; ****$p < 0.0001$ *vs.* [standard diet]; two-tailed Student's *t*-test). *Scale bars* = 50 μm (d). *Abbreviations*: cp, cortical plate; IZ, intermediate zone; MZ, marginal zone; PND, postnatal day; VZ/SVZ, ventricular zone/subventricular zone. Raw data and statistics were included in the Source Data File.

Diet batch	Standard diet	Experimental diet
Product name	824050	823106
Batch n.		37399
date of manifacture	09.02.05	18.08.14
crude fat (CF)	10.00%	34.2%
Kcal/g fresh	3.68%	5.13%
Kcal/g 10% H20	3.47%	4.81%
Vitamin E	74.09 iu/Kg	197.4%
C18:1 (n6)cis cis-12-Octadecanoic acid	n.r.	< 0.01% CF
C18:1 (n6)trans trans-12-Octadecanoic acid	n.r.	n.r.
C18:1 (n3)cis	n.r.	< 0.01% CF
C18:2 (n6)cis Linoleic acid (LA)	1.34%	10.5% CF
C18:2 (n6)trans Trans Linolelaidic acid	NA	0.2% CF
**C18:3 (n3)cis Aplha-Linolenic acid (ALA)**	**0.23%**	**3.60% CF**
C18:3 (n6)cis Gamma Linoleic acid	n.r.	0.1% CF
C18:4 (n3)cis Stearidonic acid	n.r.	1.2% CF
C20:2(n6)cis cis-11,14-Eicosadienoic acid	n.r.	0.8% CF
C20:3 (n3)cis cis-11,14,17-Eicosatrienoic acid	n.r.	0.3% CF
C20:3 (n6)cis cis-8,11,14-Eicosatrienoic acid	n.r.	0.2% CF
C20:4 (n3)cis cis-8,11,14,17-Eicosatetraenoic acid	n.r.	0.8% CF
C20:4 (n6)cis Arachidonic acid (AA)	0.01%	0.4% CF
**C20:5 (n3)cis Eicosapentenoic acid (EPA)**	**n.r.**	**4.6% CF**
C22:2 (n6)cis Docosadienoic acid	n.r.	< 0.1% CF
C22:3 (n3)cis	n.r.	0.1% CF
C22:4 (n6)cis Docosatetraenoic acid	n.r.	0.1% CF
C22:5 (n6)cis cis-4,7,10,13,16 Docosapentaenoic acid	n.r.	0.2% CF
C22:5 (n3)cis Docosapentaenoic acid (DPA)	n.r.	1.7% CF
C22:6 (n3)cis Docosahexaenoic acid (DHA)	n.r.	6.0% CF
C14:1 (n5) cis-9-Myristoleic acid	0.01%	0.1% CF
C16:1 (n7) cis-9-Palmitoleic acid	0.07%	3.7% CF
C18:1 (n9) cis Oleic acid	0.7%	9.13% CF
C12:0 (n3) cis Lauric acid	0.07%	< 0.1% CF
C14:0 (n5) cis-9-Myristic acid	0.13%	3.3% CF
C16:0 (n7) cis-9-Palmitic acid	0.23%	11% CF
C18:0 (n6) Stearic acid	0.14%	2.7% CF
**Omega-3 fatty acids**	**0.26%**	**18.3% CF**
**Omega-6 fatty acids**	**1.87%**	**12.5% CF**
**omega-3/omega-6 ratio**	**1:7.2**	**1:0.7**

Supplementary Table 1. Composition of the experimental and control diets used. Deviation in composition between batches of the diets did not exceed 1% per constituent. Data are from Special Diet Services (UK) and were produced by mass-spectrometry as part of the standard quality control process.

	Gene name	experimental diet vs standard diet (fold)
Proteosome / protein degradation/exosome	*Ubap21*	0,41
	Psme1	0,46
	Psmc6	0,48
	Rwdd1	0,60
	Serpina1	0,61
	Dnpep	0,71
	Ubap2	0,72
	Ncstn	0,79
	Ube213	1,34
	Ubxn1	1,36
	Npepps	1,44
	Psmd1	1,60
	Lonp1	1,61
	Ptub1	1,73
	Psmd6	2,07
	Psd11	2,56
	Psmc5	3,98

	Gene name	experimental diet *vs* standard diet (fold)
Cell proliferation/survival/apoptosis/cell cycle	*Krt5*	0,09
	Tppp3	0,26
	Manf	0,29
	Serbp1	0,36
	Lmna	0,39
	Caprin1	0,50
	Csrp2	0,56
	Erh	0,68
	Psmd13	1,25
	Fxr1	1,76
	Tsg101	1,80
	Srrt	2,00
	Ran	3,02
	Shc1	3,45

	Gene name	experimental diet vs standard diet (fold)
Translation / ribosome assembly / protein biogenesis /ER	*Rplp2*	0,22
	Pfdn2	0,26
	Rrbp1	0,27
	Pfdn6	0,29
	Rcn2	0,32
	Eef1b	0,38
	Rps25	0,40
	Fkbp3	0,47
	Rplp1	0,47
	Tma7	0,49
	Ssr1	0,50
	Eif4b	0,53
	Rpl23a	0,54
	Cnpy3	0,54
	Alad	0,55
	Tbca	0,58
	St13	0,59
	Sec23a	0,60
	Asna1	0,72
	Rpl24	1,42
	Eif2s1	1,42
	Rps26	1,46
	Rpl35	1,47
	Rpl22l1	1,47
	Kars	1,47
	Rpl7	1,49
	Nop56	1,58
	Rpl11	1,62
	Rpl13	1,69
	Gar1	1,71
	Rpl19	1,72
	Rpl17	1,73
	Rplp0	1,75
	Rps11	1,77
	Canx	1,78
	Fkbp10	1,78
	Rpl23	1,79
	Rpl6	1,88
	Ahsa1	1,90
	Hsph1	1,97
	Rpl7a	2,05
	Ppid	2,07
	Ganab	2,09
	Rpl8	2,20
	Rps2	2,30
	Rpl4	2,74

Supplementary Table 2. Proteins with levels significantly modified in the cortices of male mouse fetuses after gestational exposure to an experimental diet. Comparative analysis of *n* = 3 (ω-3 fatty acid/vehicle) with data expressed as percentage deviation from the mean control value. Only hits with statistically significant differences were included, as evaluated by the Wilcoxon signed-rank test. Colored backgrounds identify data clusters with positive/negative differences from vehicle-treated controls.

Marker	Dilution	Host	Source
CaCyBp	1:1,000 (IHC)	Rabbit	Human Protein Atlas/Sigma
Alexa Flour^{TM} 546 Phalloidin	1:500		Invitrogen
Beta III tubulin	1:1,000	Mouse	Promega
Calnexin	1:500	Mouse	Invitrogen
Calretinin	1:500	Guinea Pig	Synaptic Systems
Cofilin	1:500	Rabbit	Cell Signaling
GAP43	1:500	Rabbit	Millipore
GAPDH	1:500	Rabbit	Cell Signaling
MAP2	1:500	Guinea Pig	Synaptic Systems
NeuN	1:500	Mouse	Merck/Chemicon
S100A6 (IHC)	1:500	Rabbit	Human Protein Atlas/Sigma
S100A6 (WB)	1:500	Rabbit	Aviva System Biology
USP7	1:500	Rabbit	Bethyl Laboratories
USP9X	1:500	Rabbit	Bethyl Laboratories

Supplementary Table 3. Antibodies used for immunofluorescence histochemistry and Western blotting. Application-specific dilutions, hosts, and vendors for the antibodies specified were stated.

Gene		Primer sequence
Actb	(forward)	5'-ATGGTGGGAATGGGTCAGAAG-3'
	(reverse)	5'-TCTCCATGTCGTCCCAGTTG-3'
Aldh1l1	(forward)	5'-AGCTGTGCCCTGAGTAATGT -3'
	(reverse)	5'-GCACAGCTTTGTTGAGGTCA -3'
Atf4	(forward)	5'-ATGGCCGGCTATGGATGAT-3'
	(reverse)	5'-CGAAGTCAAACTCTTTCAGATCCATT -3
Atf6a	(forward)	5'-GGACGAGGTGGTGTCAGAG -3'
	(reverse)	5'-GACAGCTCTTCGCTTTGGAC -3'
CaCyBp	(forward)	5'-GGTTGCTCCTCTTACAACAGG-3'
	(reverse)	5'-TGACCTCTCTGTGAAGTGCA-3'
Chop	(forward)	5'-CCAACAGAGGTCACACGCAC -3'
	(reverse)	5'-TGACTGGAATCTGGAGAGCGA -3'
Gapdh	(forward)	5'-AACTTTGGCATTGTGGAAGG -3'
	(reverse)	5'-ACACATTGGGGGTAGGAACA -3'
Grp78	(forward)	5'-ACCCTTACTCGGGCCAAATT-3'
	(reverse)	5'-AGAGCGGAACAGGTCCATGT-3'
Map2	(forward)	5'-CTTTCCCCTCTGGCTTCTGA -3'
	(reverse)	5'-AGCAAGGCATCTTCTCCACT -3'
S100a6	(forward)	5'-ACTCTGGCAAGGAAGGTGAC-3'
	(reverse)	5'-GCCGACATACTCCTGGAAGT-3'
S100b	(forward)	5'-TGTCTTCCACCAGTACTCCG -3'
	(reverse)	5'-TCCAGCGTCTCCATCACTTT -3'
Tubb3	(forward)	5'-CTCAACCACCTTGTGTCTGC -3'
	(reverse)	5'-GAAGAAGTGGAGACGTGGGA -3'
Xbp1s	(forward)	5'-GAGTCCGCAGCAGGTG -3'
	(reverse)	5'-GTGTCAGAGTCCATGGGA -3'
Xbp1u	(forward)	5'-GACAGAGAGTCAAACTAACTGGG -3'
	(reverse)	5'-GTCCAGCAGGCAAGAAGGT -3'

Supplementary Table 4. Primer sequences used in this study. qPCR reactions were performed with primer pairs amplifying short fragments for each gene. Primer pairs were custom designed to efficiently anneal to homologous nucleotide sequences in mouse.

Supplementary References

1 Deloulme, J. C. et al. S100A6 and S100A11 are specific targets of the calcium- and zinc-binding S100B protein *in vivo. J Biol Chem* **275**, 35302-35310 (2000).

2 Topolska-Wos, A. M., Chazin, W. J. & Filipek, A. CacyBP/SIP--Structure and variety of functions. *Biochim Biophys Acta* **1860**, 79-85 (2016).

Supplementary Source File for Western blot images

Supplementary Figure 1d

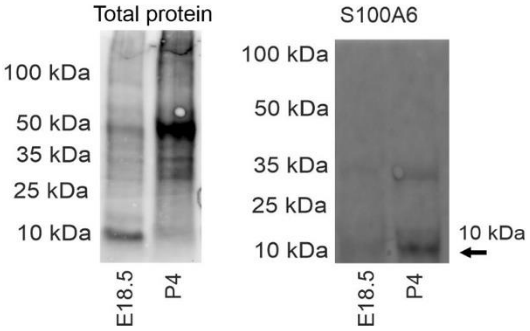

Supplementary Figure 1i

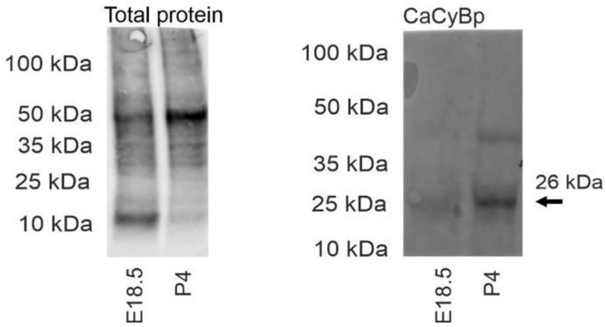

Supplementary Figure 4b

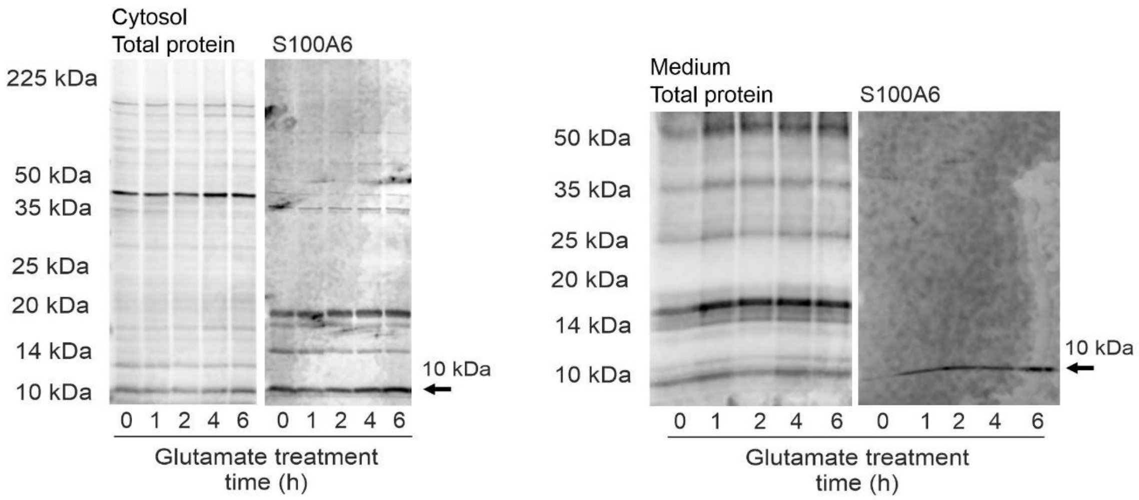

Supplementary Figure 4c

blots were cut at 40 kDa to allow for simultaneous visualization with multiple antibodies

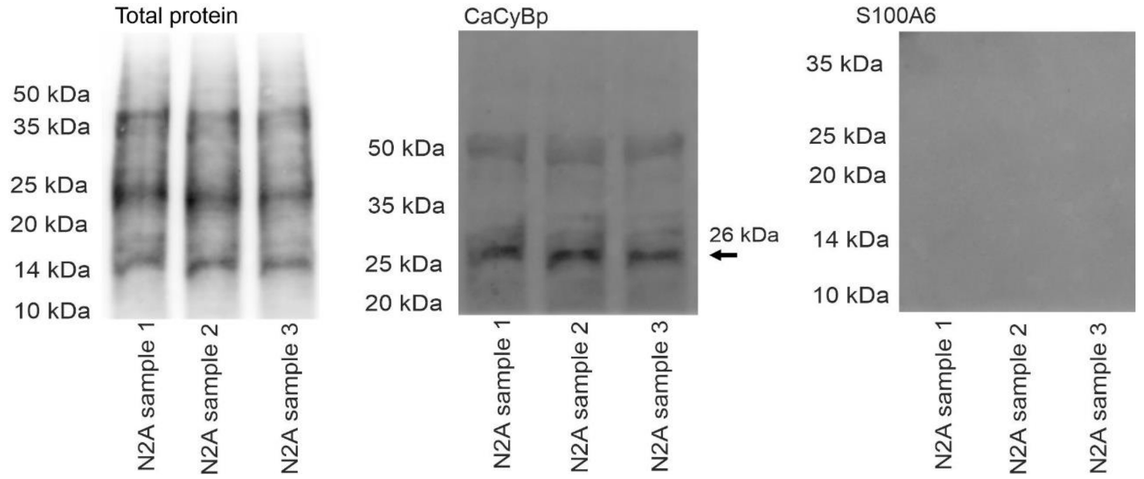

Supplementary Figure 4h

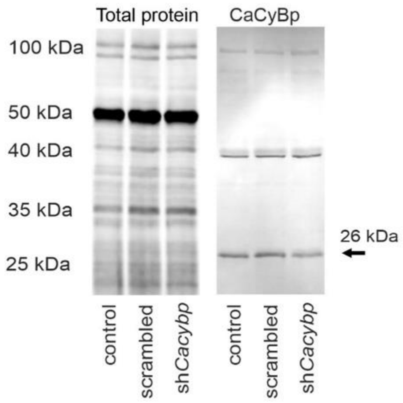

Supplementary Figure 5b

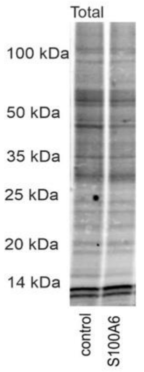

Supplementary Figure 5c

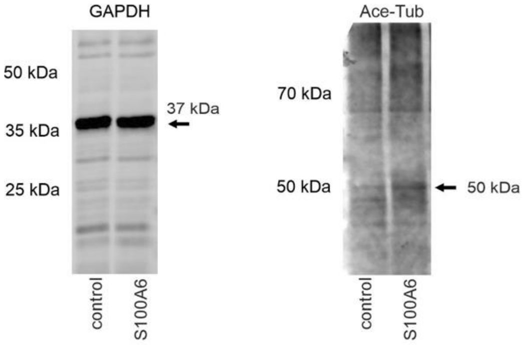

Supplementary Figure 5d

blots were cut to allow for simultaneous visualization with multiple antibodies

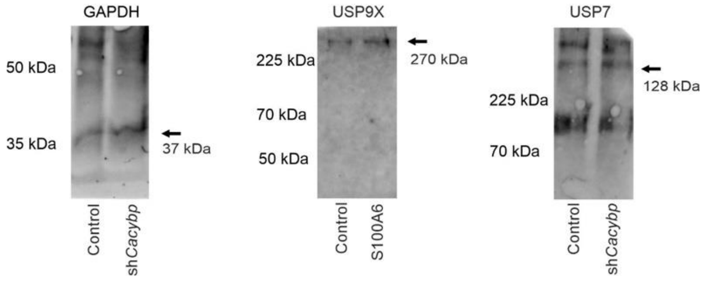

Supplement: Supplementary file 1 — Supplementary Information [file 41467_2025_64405_MOESM1_ESM.pdf]
